# 

**DOI:** 10.1192/bjb.2023.12

**Published:** 2024-04

**Authors:** Gordon Barclay

**Affiliations:** is a retired NHS consultant psychiatrist in private practice, Glasgow, UK. Email: dr@gordonbconsulting.com

**Figure:**
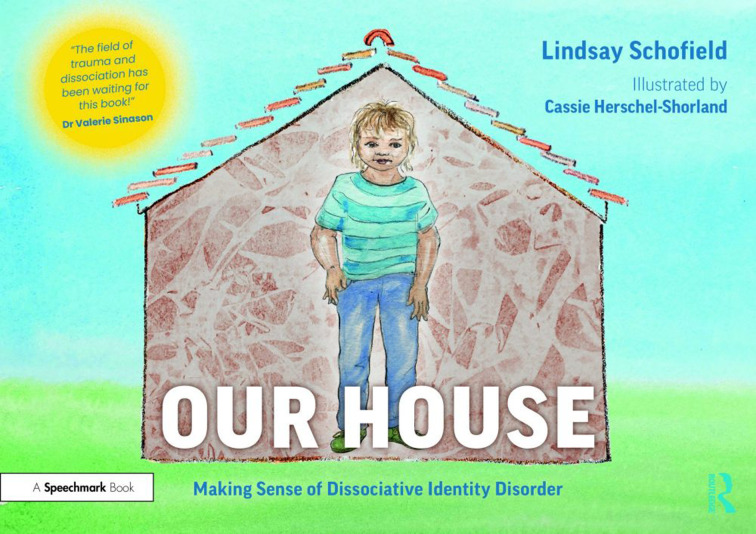

Lindsay Schofield, a consultant psychotherapist with deep experience of working with dissociative presentations and with patients with DID (dissociative identity disorder), has written two companion volumes which I wish had been to hand as I started out as a consultant psychiatrist trying to get to grips with the protean phenomenology of dissociation. In the first, through skilful collaboration between narrative and illustration (by Cassie Herschel-Shorland), sense really is made, referring to the subtitle of the book, of DID in a manner truly accessible to both patient and clinician. In the second volume, after an extended section offering more detailed commentary on each of the pictures in the first volume, other sections describe in a commendably clear and practically useful manner ‘what is trauma’, looking at the phenomenology and presentations of dissociation as a continuum, before considering the general principles of treatment, with further chapters devoted to ‘supporting practitioners’ and ‘supporting recovery’.

One of the most useful chapters in this excellent guidebook to DID, which together with the volume which it accompanies deserves a place in every psychiatric library in the land, is the final chapter entitled ‘additional resources’, which is impressively thorough as an overview and, like the rest of the book, beautifully and clearly laid out.

Although the strikingly brief entry on what was then called ‘multiple personality disorder’ (F44.81) in ICD-10 stated ‘This disorder is rare…’, the nosological landscape is not fixed, and that assertion is contradicted by evidence from many population studies conducted internationally, suggesting that the prevalence of DID in the general population is actually greater than that of schizophrenia or bipolar affective disorder. Although still controversial in some quarters, the challenge posed by the diagnosis of DID should surely be that we become more radically phenomenological in our engagement with our patients, being more willing to listen and learn from them with an open mind, especially when they might seem to be leading us into unfamiliar territory.

Lindsay Schofield’s two volumes offer an invaluable *vade mecum* for clinicians in contemporary psychiatry and psychotherapy faced with the challenge of engaging with DID in the way that the many patients with this condition deserve. My other recommendation would be to go back to re-read Freud and Breuer's classic case history of Anna O. Having perhaps perused these two enlightening volumes beforehand.

